# Supplementary material for: Splice-Junction-Based Mapping of Alternative Isoforms in the Human Proteome
Source: Cell Rep. Author manuscript; Available in PMC 2020 Jan 15. (PMC6961840; doi:10.1016/j.celrep.2019.11.026)
Supplement: 2 [file NIHMS1546469-supplement-2.zip › DF1/PXD010154/Esophagus/Esophagus_18_PWWP2A_RPATAASPGEGGAGEAEPEMEPIPGSEAGTDPLP.pdf]

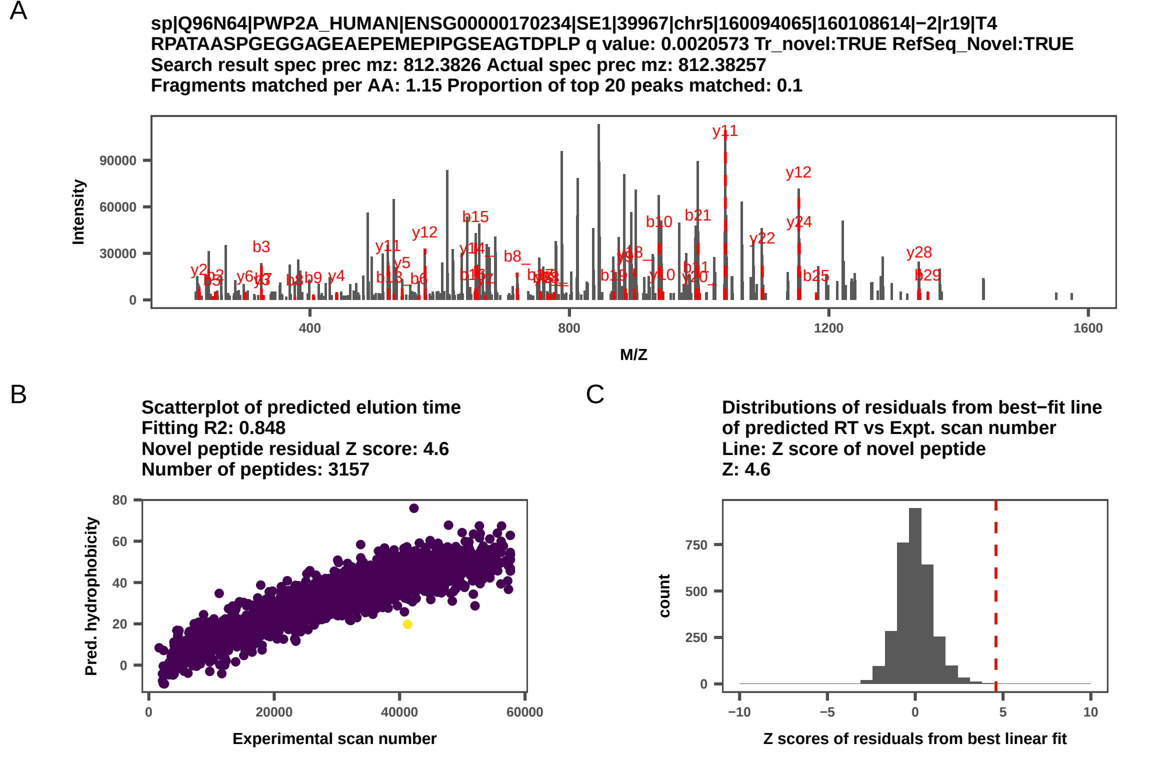
A
sp|Q96N64|PWP2A_HUMAN|ENSG00000170234|SE1|39967|chr5|160094065|160108614|−2|r19|T4
RPATAASPGEGGAGEAEPEMEPIPGSEAGTDPLP q value: 0.0020573 Tr_novel:TRUE RefSeq_Novel:TRUE
Search result spec prec mz: 812.3826 Actual spec prec mz: 812.38257
Fragments matched per AA: 1.15 Proportion of top 20 peaks matched: 0.1
Intensity
90000
60000
30000
0
400
800
1200
1600
M/Z
y11
y12
y24
y22
y28
b29
b25
b21
b10
b15
y12
y11
b3
y2
y4
b9
y5
b6
b8
y10
b19
b11
B
Scatterplot of predicted elution time
Fitting R2: 0.848
Novel peptide residual Z score: 4.6
Number of peptides: 3157
Pred. hydrophobicity
80
60
40
20
0
0
20000
40000
60000
Experimental scan number
C
Distributions of residuals from best−fit line
of predicted RT vs Expt. scan number
Line: Z score of novel peptide
Z: 4.6
count
750
500
250
0
−10
−5
0
5
10
Z scores of residuals from best linear fit
